# Looking Sideways in Capsule Endoscopy: Why Wider Views Still Need Stronger Evidence

**DOI:** 10.1002/hsr2.73101

**Published:** 2026-09-15

**Authors:** Ervin Toth, Wojciech Marlicz, Anastasios Koulaouzidis

**Affiliations:** ^1^ Department of Gastroenterology Skåne University Hospital, Lund University Malmö Sweden; ^2^ Department of Gastroenterology Pomeranian Medical University Szczecin Poland; ^3^ Endoklinika, The Center for Digestive Diseases Szczecin Poland; ^4^ Department of Clinical Research University of Southern Denmark (SDU) Odense Denmark; ^5^ Department of Surgery, SATC‐C Odense University Hospital & Svendborg Hospital Svendborg Denmark

To the Editor,

We read with interest the systematic review and meta‐analysis by Saadati et al. comparing panoramic‐view and forward‐view capsule endoscopy (CE) in suspected small‐bowel (SB) bleeding [1]. The authors should be commended for addressing a clinically relevant question in an area where comparative evidence remains limited. More than a decade ago, Koulaouzidis and Dabos asked whether “looking forward” was necessarily the best approach in CE [2]. That question remains pertinent. Nonetheless, the converse is equally important: looking sideways, or panoramically, is not automatically better unless it improves clinically meaningful outcomes. Wider mucosal visualisation is an attractive optical concept, but it should not be conflated with proven diagnostic accuracy, improved therapeutic decision‐making, or better patient outcomes [3, 4].

The principal limitation is that the review largely analyses diagnostic yield (DY) rather than diagnostic accuracy. DY is a pragmatic detection metric influenced by patient selection, bleeding phenotype, timing of CE, bowel preparation, reader behaviour, lesion classification, and denominator choice [5, 6, 7]. It is not equivalent to sensitivity, specificity, false‐negative rate, lesion‐level confirmation, therapeutic yield, rebleeding, or patient‐level outcome [5]. This distinction is especially important because DY was not defined uniformly across the included studies. Saadati et al. acknowledge variation in lesion classification, anatomical location, and denominator [1]. Some studies counted P1 and P2 lesions, whereas others focused only on P2 lesions; some used all recruited patients as the denominator, whereas others used only completed examinations [3, 4]. Pooling such definitions may generate a statistically tidy estimate, but the clinical meaning of that estimate remains blurred.

The interpretation of the non‐significant pooled DY result also requires some restraint. The pooled risk difference was close to zero, but the confidence interval remained compatible with clinically relevant absolute differences in either direction [1]. This does not establish equivalence or non‐inferiority. A conclusion that panoramic‐view and forward‐view CE are “comparable” requires a prespecified equivalence margin and adequate precision around that margin [8]. In the absence of such a framework, the safest conclusion is that superiority was not demonstrated. By contrast, the most consistent practical difference is operational rather than diagnostic: reading time was longer with panoramic‐view CE [1, 4]. This matters in real‐world capsule services. A difference of several minutes per case becomes important at scale, particularly in high‐volume units [7]. Since reading time is measured in minutes across studies, a pooled mean difference would be more clinically useful for clinicians and service planners than a standardized mean difference alone.

The definition of completion also deserves greater granularity. For CE systems with on‐board data storage, completion should not be defined solely by capsule ingestion or caecal visualisation. It also depends on capsule excretion, patient retrieval and return of the device, successful data download, and generation of an interpretable video [3, 9]. These device‐specific steps should ideally be reported separately as caecal completion, capsule recovery, data‐export success, technical failure and interpretable‐examination rate. The evidence base remains fragile. Only five studies were included; two were conference abstracts, and the quality scores were low [1]. Formal Egger and Begg tests are also difficult to interpret with only four or five studies and should not reassure readers that publication bias is absent [10]. In addition, the review would have benefited from a broader evidence map. Regulatory and trial‐registry sources, previous device‐comparison systematic reviews, and large grey‐literature datasets may not all be suitable for quantitative pooling, but they should be identified and transparently excluded or discussed when reviewing a niche device field. Accordingly, the most defensible conclusion is not that panoramic‐view CE has comparable diagnostic reliability to forward‐view CE. Rather, current comparative evidence does not demonstrate superior DY for panoramic‐view CE in suspected SB bleeding, while suggesting a longer reading burden and additional workflow considerations [1, 3, 4].

Future studies should use standardised P2 SB lesion definitions, intention‐to‐diagnose denominators, blinded independent reading, lesion confirmation where feasible, therapeutic yield, rebleeding outcomes, patient‐reported burden of capsule retrieval, reading‐time cost and health‐economic endpoints [5, 6, 7, 8, 9]. Until then, the lesson remains familiar: in CE, the direction of view is important, but validated clinical value matters more.

## Author Contributions

Anastasios Koulaouzidis conceived the letter, reviewed the source article and supporting literature, developed the main methodological critique, and drafted the manuscript. Ervin Toth and Wojciech Marlicz contributed clinical and methodological interpretation, critically revised the manuscript for important intellectual content, and approved the final version. All authors reviewed and approved the submitted manuscript and agree to be accountable for all aspects of the work.

## Funding

The authors have nothing to report.

## Disclosure

Artificial intelligence tools (ChatGPT, OpenAI, San Francisco, CA, USA) were used to assist with language editing and to support verification of selected statistical calculations. No AI‐generated data, analyses, tables, or figures were included. The authors take full responsibility for the accuracy, integrity, interpretation, and final content of the letter.

The views and opinions expressed herein are personal to the authors and do not represent the official positions of any institutions, professional societies, or organizations with which the authors are affiliated or holds leadership roles.

## Ethics Statement

This correspondence did not involve new human participants, human tissue, animals, identifiable patient data, or the generation or analysis of new data. Ethical approval and informed consent were therefore not required.

## Conflicts of Interest

A.K. and W.M. are or have been guest editors for MDPI journals. A.K.,and A.K., and W.M. are section board members of *Diagnostics* and *Journal of Clinical Medicine*. A.K. is Associate Editor of *Annals of Gastroenterology* and *Therapeutic Advances in Gastrointestinal Endoscopy* and is immediate past president of the iCARE group. W.M. is Co‐Editor‐in‐Chief of *Przeglad Gastroenterologiczny/Gastroenterology Review*, President of the Polish Society of Gastroenterology, Chair of Train the Trainers and member of the Governing Council of the World Gastroenterology Organisation, Country Representative for Poland of the European Lifestyle Medicine Organization, and Country Coordinator for Poland of the HpEuReg Registry. W.M. is shareholder and co‐founder of Sanprobi sp. z o.o. and Endoklinika sp. z o.o., and has received speaker honoraria from Ferring, Alfasigma, Novo Nordisk, and Polpharma. A.K. is co‐founder/shareholder of AJM Med‐i‐caps Ltd, has been co‐founder/director of iCERV Ltd, and has served as a consultant for Jinshan Science & Technology Ltd. A.K. has received research support from Given Imaging Ltd under the auspices of the European Society of Gastrointestinal Endoscopy, and from IntroMedic/SynMed; consultancy fees from Jinshan; lecture honoraria from Covidien/Medtronic, Jinshan, Dr Falk Pharma UK, Ferring, Aquilant, and Almirall; and educational travel support from Jinshan, Dr Falk Pharma UK, Ferring, Aquilant, and Almirall. A.K. has also participated in advisory board activities for Tillots, Ankon, and Dr Falk Pharma UK. E.T. has received research support from AnX Robotica; research support and lecture honoraria from Jinshan Science & Technology Ltd and Norgine; and consultancy and lecture fees from Medtronic and Olympus.

## Transparency Statement

The lead and corresponding author, Anastasios Koulaouzidis, affirms that this manuscript is an honest, accurate, and transparent account of the work being reported; that no important aspects of the work have been omitted; and that any discrepancies from the work as planned have been explained.

## Data Availability

Data sharing is not applicable to this article as no new data were created or analysed for this correspondence.
